# Longitudinal Vitamin D Deficiency Among Malaysian Pregnant Women and Its Correlation With Neonatal Serum 25-Hydroxyvitamin D Levels

**DOI:** 10.3389/fpubh.2021.654292

**Published:** 2021-06-29

**Authors:** Muzaitul Akma Mustapa Kamal Basha, Hazreen Abdul Majid, Nuguelis Razali, Aswir Abd Rashed, Hussin Muhammad, Abqariyah Yahya

**Affiliations:** ^1^Department of Social and Preventive Medicine, Faculty of Medicine, University of Malaya, Kuala Lumpur, Malaysia; ^2^Department of Special Care Nursing, Kulliyyah of Nursing, International Islamic University Malaysia, Kuantan, Malaysia; ^3^Department of Nutrition, Faculty of Public Health, University of Airlangga, Surabaya, Indonesia; ^4^Department of Obstetrics & Gynaecology, Faculty of Medicine, University of Malaya, Kuala Lumpur, Malaysia; ^5^Nutrition, Metabolism & Cardiovascular Research Centre, Institute for Medical Research, Kuala Lumpur, Malaysia; ^6^Herbal Medicine Research Centre, Institute for Medical Research, Kuala Lumpur, Malaysia

**Keywords:** vitamin D deficiency, cord blood, pregnancy, neonates, 25(OH)D

## Abstract

**Objective:** This study aimed to investigate the longitudinal relationship between maternal vitamin D concentrations during pregnancy and neonatal vitamin D concentrations at birth.

**Materials and Methods:** A prospective cohort of 236 healthy pregnant women from various ethnicity in early pregnancy (≤20 weeks of pregnancy) was followed at late pregnancy (28–40 weeks of pregnancy) and birth. Maternal serum 25-hydroxyvitamin D (25(OH)D) was assessed at early pregnancy (baseline) and late pregnancy, while neonatal cord serum 25(OH)D at birth. General estimating equations (GEE) were used to analyze the longitudinal association of maternal serum 25(OH)D levels during pregnancy and neonatal cord serum 25(OH)D levels at birth with adjusting for the time exposure, maternal weight gain, ethnicity, and skin type.

**Results:** The results showed that the prevalence of vitamin D deficiency (25(OH)D <50 nmol/L) was at 89.9, 92.2, and 96.1% in early, late pregnancy and in neonatal cord serum, respectively. The GEE analysis showed a trend that longitudinal vitamin D deficiency during pregnancy leads to lower vitamin D concentrations in neonatal cord blood (RR = 1.17; 95% CI (1.05–1.36); *p* = 0.04).

**Conclusion:** Longitudinal vitamin D deficiency during pregnancy leads to vitamin D deficiency in neonates at birth. A further trial is needed to affirm this association.

## Introduction

Malaysia is rich in sunlight (equatorial climate), a primary source of vitamin D. However, a study by Woon et al. (1) among Malaysian pregnant women in the third trimester of pregnancy showed that 92.0% had vitamin D deficiency (25(OH)D <50 nmol/L). Lee et al. (2) reported that more than 70% of third-trimester pregnant women in the capital of Malaysia, Kuala Lumpur had vitamin D deficiency (25(OH)D <50 nmol/L). A study conducted by Mohammed et al. (3) in a rural area in Malaysia had shown that 60.0% of pregnant women in the second trimester had vitamin D deficiency (25(OH)D <50 nmol/L). However, most of these studies are prevalence studies.

Vitamin D levels from a single time point of measurement do not have the explicit vitamin D level throughout pregnancy (3, 4). Throughout the pregnancy course, vitamin D status could vary. Different investigators reported different outcomes of 25(OH)D levels, either a decline or an increase, or absence of changes across the pregnancy course (5, 6). Nevertheless, these divergent data were from Western populations. This phenomenon could be attributed to seasonal sampling. Those living in areas with insufficient sunlight (a primary source of vitamin D) were at risk of vitamin D deficiency (7, 8). Furthermore, data on vitamin D status in neonates and infants, particularly in Asian countries, including Malaysia, are scarce (9). In Malaysia, the literature is limited to adolescent and adult populations (10–13).

Attention must be given to pregnant women as vitamin D deficiency during pregnancy has multiple adverse obstetric and child health outcomes (4, 5, 14). Vitamin D deficiency during pregnancy has been reported to affect fetal growth and development, which can be detrimental and may translate into pathology in later child health (9, 14, 15). Vitamin D deficiency during pregnancy is also associated with neonatal vitamin D levels (16–18). However, the associations have not been entirely consistent. Results from a small study of 58 pregnant women and their neonates in Beijing, China, found that maternal serum vitamin D concentrations had a significant positive correlation with cord serum vitamin D concentrations (*r* = 0.89, *p*-value < 0.001) (19). In contrast, a study conducted in India reported no significant difference (*p* = 0.820) in vitamin D concentration levels between paired maternal and umbilical cord blood samples, potentially due to the small number of participants (*n* = 20) (20).

Given the facts above, the present study has (1) evaluated longitudinal maternal 25(OH)D levels across the pregnancy progression (2) determined the longitudinal association of maternal 25(OH)D levels and neonatal 25(OH)D levels at birth via cord serum.

## Materials and Methods

### Study Design

This study was a 2-year prospective cohort study conducted between November 2017 to March 2019 at a teaching hospital (University Malaya Medical Center (UMMC) in an urban district, Kuala Lumpur, in Malaysia. The sample size was calculated using an Open Source Epidemiologic Statistics for Public Health (OpenEpi) software based on the risk difference previous study finding (21).

The inputs used were a two-sided significance level (set at 95%), power (1-beta, % chance of detecting) was set at 80%, and risk difference was set at 14. The risk difference was derived from the risk difference of the mother in low quartile 25(OH)D level during pregnancy with low quartiles of 25(OH)D in cord blood compared to the mother in high quartile 25(OH)D level during pregnancy with high quartiles of 25(OH)D in cord blood (21). After considering a 20% attrition rate, the required sample size was about 236 pairs of mother-neonate. A convenience sampling method was adopted to recruit the participants. Given the time constraint, lack of human resources, and the study population's nature in the study setting (more referral cases), random selection was not possible to apply.

### Research Ethics

This study was approved by the Medical Ethics Committee of the UMMC, Kuala Lumpur [MREC ID NO: 2017828-5528].

### Study Participants

Healthy pregnant women with gestational age ≤20 weeks of gestation and singleton of pregnancy were invited to participate in this study if they met the eligibility criteria: being Malaysian, aged 18–45 years old, no history of medical illness (such as chronic renal disease, bone disease, parathyroid disorders, and psychiatric disorders), singleton and ≤20 weeks of pregnancy. A thorough explanation of the study objectives and how the researcher would conduct the study was given to the eligible pregnant women. Informed consent was obtained from each participant before they participated in the study.

The participants were assessed at three times points: early pregnancy (≤20 weeks of pregnancy) (baseline), late pregnancy (28–40 weeks of pregnancy), and at birth (cord blood). The proportion of pregnant women in the first trimester was small than the other trimesters in our study setting. The majority of them were in late second or third trimesters. Thus, this study applied early (≤20 weeks of pregnancy) and late pregnancy (28–40 weeks of pregnancy) instead of trimesters because of the nature of the study population in the study setting. The participants who were accomplished by assisted reproductive techniques such as IUI, IVF, multiple pregnancies, current use of illicit drugs (defined as any use in the last 6 months), and delivered preterm were excluded from this study.

**Figure 1:**
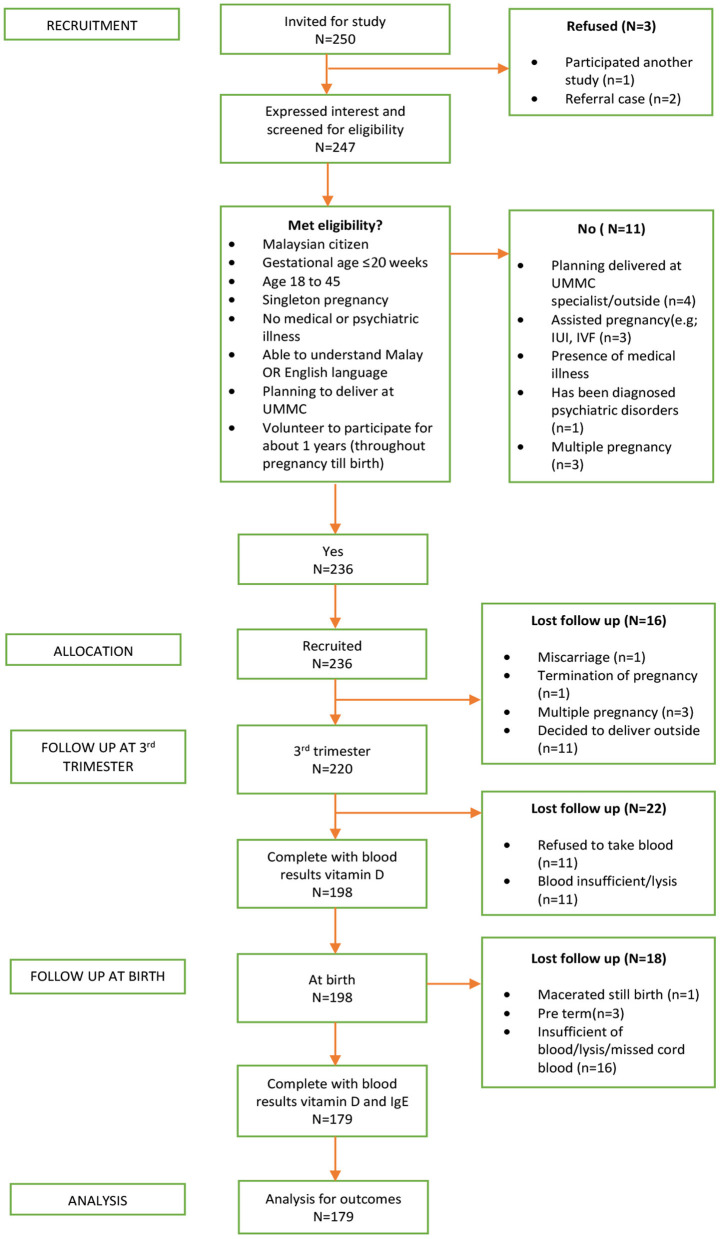
Recruitment flow diagram of the prospective cohort study.

A total of 236 pregnant women initially participated in the study. Nevertheless, the follow-up was completed by only 179 mother-neonate pairs and included in the final analysis, yielding a 76% completion rate. The reasons for participants' loss to follow-up were a miscarriage, delivered outside the study setting, preterm delivery, and insufficient or blood samples lysed. The detail of the derivation of the study sample was presented in Figure 1.

### Serum 25(OH)D Levels in Early and Late Pregnancy (Maternal Serum) and at Birth (Cord Serum)

Maternal venous blood samples were collected into plain test tubes by a trained nurse upon recruitment to assess the 25(OH)D of the mothers in early pregnancy. Then, the 25(OH)D of the mothers were reassessed at late pregnancy during their antenatal visit appointment. Meanwhile, cord blood samples (free flow to avoid lysis) were collected to assess the 25(OH)D of neonates at birth. All the blood samples were kept at room temperature for 30–60 min to allow for clotting. Then, they were centrifuged (at 3,500 rpm for 10 min). The plasma was separated and stored at −80°C on the same day until analyses.

A High-Performance Liquid Chromatography (HPLC) was chosen to measure the vitamin D levels as it is considered a standard gold method to measure the 25(OH)D. It has greater specificity and increased precision compared with immunoassays (22). Currently, the HPLC is not used routinely in many local clinical settings. The 25(OH)D analysis in this study was conducted at Pantai Premier Pathology Pte Ltd. Laboratory, Kuala Lumpur using the HPLC with a C8 column coupled to an API 3200 Q-trap mass spectrometer (MS, AB SCIEX Framingham, MA, USA). The limit of quantification was ~2nmol/L.MS) (standard gold method) (23, 24). The coefficient of variation of the test was 0.58% (22). The Pantai Premier Pathology Laboratory Pte Ltd maintained a Quality Management System according to the requirements of ISO 9001: 2000 (Company registration: 438067-X).

Vitamin D status was based on plasma 25(OH)D concentrations and categorized as vitamin D sufficient (25(OH)D ≥50 nmol/L) or deficiency (25(OH)D <50 nmol/L), according to the Institute of Medicine (IOM) recommendations (23, 24). We also tested the thresholds of 25(OH)D <25 nmol/L and <75 nmol/L (23, 24).

### Covariates Assessment

The maternal sociodemographic, obstetrics status, skin type color, and anthropometry measurements were recorded accordingly. Maternal body weight (kg) and height (m) was measured upon enrolment using a calibrated integrated Stadiometer (Seca703s Germany), and pre-pregnancy body weight was obtained from the participants by self-reported. Early gestational weight gain (≤20 weeks of pregnancy) was calculated based on gestational weight gain recommendations by the Institute of Medicine (IOM), dependent upon maternal pre-pregnancy BMI. According to IOM guidelines, there are three classifications of gestational weight gain regardless of age, parity, smoking history, race, and ethnic background, which are inadequate, adequate, and excessive (25). The Fitzpatrick Skin Type Chart Measurement (FSTCM) was used to measure the participants' skin color (26, 27).

The past month's daily vitamin D intake was estimated using a validated semi-quantitative food frequency questionnaire (FFQ vitamin D/My) (28). The maternal daily vitamin D intake was calculated using the Nutritionist ProTM Diet Analysis software (Axxya Systems, Woodinville, WA, USA). The Singapore Energy and Nutrient Composition of Food Database is used as a reference for vitamin D intake.

### Statistical Analysis

Statistical analysis was performed using the SPSS software version 22.0 (IBM, Armonk, NY). The data were cleaned and checked for coding errors using a random and consistent method. Descriptive statistics were used for participants' characteristics. The descriptive results and presented using means and standard deviations (SDs) for continuous variables with normal distributions, medians, and interquartile range (IQRs) for continuous variables without normal distributions and relative frequencies (%) for categorical variables. Statistical comparisons of baseline data between the completer and dropped out participants were performed and found no statistically significant difference between completers and dropped out, except skin color type. Likewise, no difference was found between these two groups in serum 25(OH)D concentrations at baseline.

This study's potential confounding factors were determined by comparing the model without additional factors and additional factors. The finding between the two OR is ≥10%; confounders were considered present. After testing, the ORs for all the potential confounders in this study were <10%, showing no confounding effects. However, variables of time exposure, maternal weight gain, ethnicity, and skin type were included in longitudinal analysis. Longitudinal associations between vitamin D concentrations during pregnancy and vitamin D concentration in cord blood were presented as relative risks (RR) and 95% confidence interval (95%CI) using generalized estimating equations (GEE). The distribution of the outcome variable (neonatal cord serum 25(OH)D) was set as “poisson” and the link function as “logit.” The working correlation structure was “independent” as there was no missing data, and the follow-up period of each participant was similar. Other working correlation structures were tested, but the independent working correlation structure gave the lowest quasi-likelihood under the independence model criterion (QIC) value. Only those with complete data throughout pregnancy until birth were included. The level of significance was set at <0.05.

## Results

### Sample Characteristics

The mean age of mothers was 31.5 ± 4.6 years (range: 21–45 years). Majority of them were Malay (77.1%, n/N = 138/179), married (99.4%, n/N = 178/179), had a high level of education (college degree or higher) (72.6%, n/N = 130/179) and formally employed (87.2%, n/N = 156/179), with 55.1% (n/N; 86/156) being professionals. One mother claimed to be a former smoker, while others were non-smokers. The average gestational age at recruitment was 16.25 weeks ± 1.60(SD), with 69.3% (n/N = 124/179) of mothers being multigravida and the average parity being 3 ± 1.58 (SD). Nearly half of the mothers (46.9%, n/N = 84/179) had light brown (type IV) skin color based on the Fitzpatrick Skin Type Chart Measurement (FSTCM), followed by 22.9% (n/N = 41/179) olive (type III), 21.2% (n/N = 38/179) fair (type II), 6.7% (n/N = 12/179) dark brown (type V) and 2.2% (n/N = 4/179) light white (type I). Anthropometry results revealed 14.0% (n/N = 25/179) classified as overweight and 38.0% (n/N = 68/179) obese prior to pregnancy. The average daily intake of vitamin D among mothers was 5.30 ± 2.22 micrograms at baseline (in the early pregnancy ≤20 weeks of pregnancy). Only 11.1% of mothers in early pregnancy obtained supplements containing vitamin D (see Table 1). Milk and milk products were the most common vitamin D intake sources, followed by meat and meat products, fish and fish products, eggs, cereal and cereal products, and beverages (see Table 2).

**Table 1:** Baseline characteristics of pregnant women who completed the study (*n* = 179) and those who dropped out (*n* = 57).

**Variables**	**Completed**	**Dropped out**	**Chi^**2**^/t**	***P*-value**
Maternal age (years) *Mean ± SD*	*31.18 ± 4.46*	*31.67 ± 4.33*	−0.74	0.46
Maternal ethnicity:				
• Malay	138 (75.0%)	46 (25.0%)	0.91^f^	0.87
• Chinese	25 (78.1%)	7 (21.9%)		
• Indian	12 (75.0%)	4 (25.0%)		
• Others	4 (100.0%)	0 (0.0%)		
Marital status				
• Married	178 (75.7%)	57 (24.3%)	0.32^f^	1
• Unmarried	1 (100.0%)	0 (0.0%)		
Maternal educational level:				
• Lower	49 (73.1%)	18 (26.9%)	0.38^a^	0.621
• Higher	130 (76.9%)	39 (23.1%)		
Maternal employment status:				
• Working	156 (76.5%)	48 (23.5%)	0.32^a^	0.611
• Not working	23 (71.9%)	9 (28.1%)		
Household income:				
• < RM4000	57 (79.2%)	15 (20.8%)	0.62^a^	0.51
• ≥RM4000	122 (74.4%)	42 (25.6%)		
Maternal smoking status:				
• Non-smoker	178 (76.7%)	54 (23.3%)	5.57^f^	0.051
• Former smoker	1 (33.3%)	2 (66.7%)		
• Current smoker	0 (0.0%)	1 (100%)		
Weight during enrolment (kg) (*Mean ± SD)*	*63.72 ± 14.07*	*61.76 ± 13.91*	0.95	0.351
Weight before pregnancy (kg) *(Mean ± SD)*	*60.45 ± 14.25*	*58.87 ± 14.30*	0.75	0.45
BMI during enrolment (kg) *(Mean ± SD)*	*25.60 ± 5.34*	*25.09 ± 5.64*	0.63	0.53
BMI before pregnancy (kg) *Mean ± SD*	*24.28 ± 5.44*	*23.90 ± 5.70*	0.46	0.651
Early gestational weight gain (0.5–0.75 kg/month)				
• Inadequate	70 (73.7%)	25 (26.3%)	1.30^a^	0.521
• Normal	28 (71.8%)	11 (28.2%)		
• Excessive	81 (79.4%)	21 (20.6%)		
Gestational age at enrolment (*Mean ± SD)*	*16.22 ± 1.60*	*16.02 ± 1.76*	0.84	0.4
Type of pregnancy				
• Primigravida	55 (73.3%)	20 (26.7%)	0.39^a^	0.54
• Multigravida	124 (77.0%)	37 (23.0%)		
Parity *(Mean ± SD)*	*2.6 ± 1.58*	*2.4 ± 1.41*	0.6	0.541
Fitzpatrick skin classification				
• Type I (light white)	4(80.0%)	1(20.0%)	32.68^f^	** <0.001**
• Type II (fair)	38(100.0%)	0(0.0%)		
• Type III (olive)	41(87.2%)	6(12.8%)		
• Type IV (light brown)	84(69.4%)	37(30.6%)		
• Type V(dark brown)	12(48.0%)	13(52.0%)		
Intake of vitamin D in food (ug) (*Mean ± SD*)	*5.30 ± 2.22*	*5.30 ± 1.02*	0.32	0.972
Serum 25(OH)D levels at baseline				
• Deficiency (<50 nmol/L)	161(76.3%)	50(23.7%)	0.23^a^	0.635
• Sufficiency (≥ 50 nmol/L)	18(72.0%)	7(28.0%)		

*Completion rate of study is 76%*.

*Chi^2^, Chi-square test; t, T-independent test*;

a *Pearson chi-square test*;

f *Fisher's exact test*.

*Italic values = Mean ± SD; Bold = Significant <0.05*.

**Table 2:** Contribution of food items toward the daily mean intake of vitamin D over the past 1 month among study participants (*n* = 179).

**Food items**	**Contribution (%)**
Milk and milk products	30.0
Fresh milk (Full cream/low-fat/flavored)	13.8
Maternal milk powder	6.2
Milk powder (Full cream/low-fat)	2.3
Sweetened condensed milk	7.4
Cheese	1.1
Ice-cream	0.1
Butter	0.1
Meat and meat products	19.8
Chicken	9.5
Beef	4.2
Beef sausage	2.0
Pork	4.0
Cow liver	0.1
Fish and fish products	17.6
Indian mackerel	5.1
Eastern little tuna	5.3
Prawn	4.5
Spanish mackerel	2.3
Salmon	0.2
Anchovy	1.0
Canned sardine	0.1
Canned tuna	0.1
Canned mackerel	0.1
Eggs	9.4
Cereal and cereal products	9.2
Bread	5.8
Cereal drinks	2.1
Pancake	0.1
Waffle	1.2
Others	1.7
Margarine	0.2
Mushroom	0.2
Mashed potatoes	0.8
Lasagna, spaghetti with cheese	0.5
Beverages	1.2
Fortified soy drinks	1.1
Glucose drink fortified with vitamin D	0.1
Supplements containing vitamin D	11.1

### Maternal 25(OH)D Concentration at Early and Late of Pregnancy

Vitamin D deficient (25(OH)D <50 nmol/L) were found to be 89,9% (n/N = 161/179), with 29,1% (n/N = 52/161) of them falling into the severe deficiency (25(OH)D <25 nmol/L) in the early pregnancy (≤20 weeks of pregnancy). The vitamin D deficiency rate was increased to 92.2% (*n* = 165/179) at late pregnancy (28–40 weeks of pregnancy). Only 10.1% (n/N = 18/179) had serum 25(OH)D ≥50 nmol/L in early of pregnancy and 7.8% (n/N = 14/179) in late pregnancy. There is no significant difference in the prevalence of maternal vitamin D deficiency across the pregnancy course (89.9 vs. 92.2%) (see Table 3).

**Table 3:** Vitamin D status in early and late pregnancy and neonatal cord serum (*n* = 179).

**25(OH)D serum concentrations**	**Early pregnancy (≤20 weeks of pregnancy)**	**Late pregnancy(28–40 weeks of pregnancy)**	**At birth (neonatal cord serum)**
	**Frequency (%)**	**Frequency (%)**	**Frequency (%)**
25(OH)D serum concentration (nmol/L) (*Mean ± SD)*	*32.51 ± 11.92*	*32.30 ± 12.98*	*31.34 ± 13.45*
Severe deficiency (<25 nmol/L)	52(29.1%)	51(28.5%)	50(27.9%)
Mild deficiency (25 – <50 nmol/L)	109(60.9%)	113(63.1%)	122(68.2%)
Insufficiency (50 – <75 nmol/L)	17(9.5%)	13(7.3%)	7 (3.9%)
Sufficiency (≥75 nmol/L)	1(0.6%)	2(1.1%)	0(0.0%)
Deficiency (<50 nmol/L)	161(89.9%)	165(92.2%)	172(96.1%)
Sufficiency (≥50 nmol/L)	18(10.1%)	14(7.8%)	7(3.9%)

*Italic = Mean ± SD*.

### Neonatal Cord Serum 25(OH)D Concentration at Birth

The descriptive analysis found 96.1% (n/N = 172/179) neonates had cord serum 25(OH)D <50 nmol/L at birth with 27.9% (n/N = 50/172) had severe deficiency (25(OH)D <25 nmol/L). Sixty-eight percent of neonates had mild deficiency (25(OH)D, 25–50 nmol/L) at birth. Only 3.9% (n/N = 7/179) of neonates in this study found to be vitamin D sufficient (25(OH)D, 50–75 nmol/L), and none had optimal (≥75 nmol/L) at birth. Overall, the prevalence of vitamin D deficiency in neonates at birth was found to be high, similar to the prevalence of mothers throughout pregnancy (see Table 3).

### Longitudinal Association Between Maternal Serum 25(OH)D Concentration Throughout Pregnancy and Neonatal Cord Serum 25(OH)D Concentration at Birth

The generalized estimating equations (GEE) analysis demonstrated a significant trend of maternal vitamin D deficiency (25(OH)D <50 nmol/L) leads to lower neonatal vitamin D concentrations at birth through the assessment of cord blood (RR = 1.17; 95% CI (1.05–1.36); *p* = 0.04) (see Table 4). Vitamin D deficiency (25(OH)D <50 nmol/L) during pregnancy increased the risk (17.0%) of neonates to have 25(OH)D deficiency at birth.

**Table 4:** Longitudinal association between vitamin D concentrations of mothers throughout pregnancy and neonates at birth (*n* = 179).

**Variables**	***B***	**Standard error**	**RR (95% CI)**	***P*-value**
25(OH)D concentrations				
• Deficiency (<50nmol/L) • Sufficiency (≥50nmol/L)	0.16	0.08	1.17 (1.05–1.36)	0.04

*Quasi Likelihood Under Independence Model Criterion (QIC) = 27.56, Corrected Quasi Likelihood Under Independence Model Criterion (QICC) = 44.18*.

*Adjusted for time exposure (early or late of pregnancy), gestational weight gains in early pregnancy, ethnicity & skin type*.

## Discussion

The present study consisted of 179 pairs of mother-neonate found a significant association between maternal vitamin D concentrations during pregnancy and neonatal vitamin D concentrations in cord blood. Vitamin D deficiency (25(OH)D <50 nmol/L) in the early & late pregnancy potentially increased risk (17.0%) of neonatal vitamin D deficiency (25(OH)D <50 nmol/L) at birth. This finding supports the previous studies where an infant born from a mother with vitamin D deficiency had low concentrations of 25(OH)D during their 1st year of life (29, 30). A study conducted in India found that 96% of the mothers had vitamin D deficiency (<50 nmol/L) during pregnancy and that 99% of their infants had vitamin D deficiency (<50 nmol/L) (29).

Meanwhile, Ariyawatkul et al. (31) reported that a significant correlation was found between maternal vitamin D serum and cord serum vitamin D levels (*r* = 0.86; *p*-value < 0.001). A recent South of India study reported that about 65% of pregnant women in the third trimester had vitamin D deficiency, and 72.6% of their infants had vitamin D deficiency (32). However, the study did not find any significant association between maternal vitamin D status during pregnancy and infants' vitamin D deficiency (*p*-value = 0.05), potentially due to small sample size (*n* = 73).

This study reported that the prevalence of vitamin D deficiency (25(OH)D <50 nmol/L) was high and increased throughout the pregnancy course. About nine out of 10 healthy pregnant women in urban Malaysia suffered from vitamin D deficiency throughout their pregnancy. Our findings verified the previous local prevalence studies whereby a high prevalence of vitamin D deficiency was reported in all trimesters (1, 4, 33, 34). The current study revealed that the prevalence of vitamin D deficiency (25(OH)D <50 nmol/L) was 89.9% in the early pregnancy (≤20 weeks of pregnancy) and 92.2% at late pregnancy (28–40 weeks of pregnancy).

This finding contradicted several previous studies that indicated that vitamin D deficiency was less severe at late pregnancy than early pregnancy (3, 35). Nevertheless, a recent study from a Danish Caucasian cohort reported a non-linear trend for 25(OH)D levels during pregnancy. They found that the 25(OH)D level peaked at week 21–34 of pregnancy and declined gradually (36). Several explanations have been proposed due to the decreasing vitamin D level throughout the pregnancy, including decreased exposure to sunshine, increasing in vitamin D-binding protein levels and changes in plasma composition (such as calcium level; adaptation of calcium metabolism due to pregnancy and the level of 1, 25(OH)_2_D) (37, 38).

Previous evidence reported although the conversion of vitamin D to 25(OH)D appears unchanged during pregnancy, the conversion of 25(OH)D to 1, 25(OH)2D during pregnancy is unique and unparalleled during life (39). During pregnancy, 25(OH)D is closely linked with 1, 25(OH)^2^D production. By 12 weeks of gestation, the serum concentrations of 1, 25(OH)_2_D are more than double that of the non-pregnant woman (40). However, such factors (i.e., sun exposure, vitamin D-binding protein receptor, and plasma composition) were not tested in our study (1, 4, 33).

During pregnancy, 25(OH)D is closely linked with 1, 25(OH)^2^D production. By week 12 of gestation, the serum concentrations of 1, 25(OH)^2^D are more than double that of the non-pregnant woman (40). It continues to rise 2–3-fold from the non-pregnant baseline, rising to over 700 pmol/L (1 pmol/L = 0.001 nmol/L), attaining levels that would be toxic due to hypercalcemia to the non-pregnant individual but are essential during pregnancy (40). Increased serum concentrations of 1, 25(OH)^2^ D potentially contributed to the decreasing 25(OH)D levels along the pregnancy course (33).

Low levels of vitamin D were hypothesized directly related to a large amount of adipose tissue in obese individuals as vitamin D is a fat-soluble vitamin. Vitamin D is postulated to be stored in the excess adipose tissue, leading to less bioavailability (41). Previous evidence also suggested that low serum 25(OH)D results from volumetric dilution of vitamin D in the large adipose stores (42). Thus, a higher amount of vitamin D is required to saturate this “reservoir,” predisposing obese individuals to insufficient serum 25OHD. This study shows that nearly half (45.3%) of the Malaysian pregnant women in an urban area had early excessive gestational weight gain. However, the current study did not test the association between a factor of gestational weight gain and vitamin D status of the mothers.

The high prevalence also was seen in neonatal cord blood at birth (96.1%). Kochar et al. (18) suggested that the high socioeconomic status is associated with vitamin D deficiency in infancy. They found that the mothers' upper and upper-middle socioeconomic status had a slightly lower prevalence of vitamin D deficiency among the neonates at birth. The present study was conducted in an urban area in Malaysia, in which most of the mothers in this study are from the upper-middle socioeconomic status. However, the present finding showed that almost all neonates had vitamin D deficiency at birth.

The present findings emphasize the need for intervention policies to prevent vitamin D deficiency during pregnancy. Food fortification and targeted vitamin D supplementation policies are recommended when the disease burden is >20%, particularly in low and middle-income countries (43). Malaysia is the upper-middle-income group of countries according to standard classifications (44). Identifying the high prevalence of vitamin D deficiency across the pregnancy and neonatal cord serum indicates that vitamin D supplementation and food fortification are needed to raise an individual's vitamin D concentrations. Ultimately reduce the risk of developing health problems such as rickets in infancy (45).

To our best knowledge, this study represents the first study in Malaysia to investigate the vitamin D status in neonatal cord blood and longitudinal maternal vitamin D status in an urban area in Malaysia. The present study adds to evidence of a high prevalence of vitamin D deficiency in pregnant women and their infants in the Asian population, mainly South-East Asia. The longitudinal approach in this study allows us to draw a causal relationship between maternal vitamin D deficiency during pregnancy leads to the lower vitamin D level in neonates at birth. Also, vitamin D levels in this study were measured using a gold standard HPLC which is more accurate than the immunoassays method.

Nevertheless, our study has some limitations to be addressed. The present findings may not represent the whole Malaysian pregnant women, but we believe that the chosen study population somehow represents the Malaysian pregnant women in urban population. Although the randomized sampling method is the gold standard in any research, it could not be adopted in the current study because of the restriction of our inclusion criteria and study time constraints; besides the nature of the study population, more referral cases in the study setting.

## Conclusion

In conclusion, vitamin D deficiency was prevalent among pregnant women during their pregnancy and neonates at birth. Longitudinal maternal 25(OH)D during pregnancy leads to lower 25(OH)D in neonatal cord blood. The present study highlights the need for a national guideline on vitamin D supplementation and treatment in pregnancy to reduce the risk of health problems in infancy. Further intervention studies are required to validate this study and identify risk reduction while correcting maternal vitamin D levels among the Malaysian population. Improving awareness among the public and health care professionals, food fortification, and targeted national programs on vitamin D supplementation are beneficial to reduce the prevalence of vitamin D deficiency in pregnancy.

## Data Availability Statement

The raw data supporting the conclusions of this article will be made available by the authors, without undue reservation.

## Ethics Statement

The studies involving human participants were reviewed and approved by Medical Ethics Committee of the UMMC, Kuala Lumpur [MREC ID NO: 2017828-5528]. The patients/participants provided their written informed consent to participate in this study.

## Author Contributions

MM, NR, HA, AY, AA, and HM conceived the study. MM and NR conducted the study. MM, HA, AA, and HM performed the laboratory analysis. MM, HA, and AY performed the statistical analysis. MM wrote the first draft of the manuscript. HA, AY, NR, AA, and HM revised the manuscript. HA, AY, NR, AA, and HM contributed to interpreting the findings. All authors reviewed and approved the final manuscript.

## Conflict of Interest

The authors declare that the research was conducted in the absence of any commercial or financial relationships that could be construed as a potential conflict of interest.
